# A longitudinal study on the mental health status of young and middle-aged orthodontic patients

**DOI:** 10.2340/aos.v85.46107

**Published:** 2026-05-28

**Authors:** Xin-yun Miao, Ying Zhang, Bin Xiong, Meng-yao Liang

**Affiliations:** a Department of Orthodontics, Affiliated Nantong Stomatological Hospital of Nantong University, Nantong, China; b Department of Nursing, The Sixth People’s Hospital of Nantong, Jiangsu, China

**Keywords:** young and middle-aged, orthodontics, mental health, influencing factors, longitudinal studies

## Abstract

**Objectives:**

To assess the mental health trajectory and its influencing factors in young and middle-aged orthodontic patients.

**Methods:**

In a longitudinal study, 154 patients were recruited from Nantong Stomatological Hospital (March 2023–October 2024). A 1:1 propensity score matching (PSM) control cohort was established to balance demographic characteristics. Data on general information, social support (Social Support Rating Scale), and psychological distress (Kessler 10 scale) were collected at eight time points from treatment initiation to 15 months. Mental health trajectories were analyzed using generalized estimating equations (GEE).

**Results:**

The GEE model indicated a significant temporal trend in mental health scores (Wald χ² = 304.51, *p* < 0.001). Significant factors included female gender (β = 6.12, *p* < 0.001), student status (β = 2.18, *p* = 0.020), higher monthly household income (>10,000 RMB (China’s currency): β = –3.51, *p* < 0.001), higher education (Bachelor’s degree or above: β = –1.89, *p* < 0.001), unmarried status (β = 3.05, *p* = 0.010), severe malocclusion (Class III: β = 1.23, *p* = 0.018), and social support (β = –0.35, *p* < 0.001), which was a protective factor.

**Conclusions:**

The mental health of young and middle-aged orthodontic patients follows a dynamic trajectory, associated with a combination of sociodemographic and clinical factors. Patients who are female, students, unmarried, or have severe malocclusion are at higher risk, whereas strong social support is protective. These findings highlight the need for timely psychological monitoring and personalized interventions for at-risk individuals throughout orthodontic care.

## Introduction

Malocclusion is a common oral health condition among young and middle-aged populations. According to the World Dental Federation [1], it not only increases susceptibility to dental caries and periodontitis but can also impair essential functions such as chewing, swallowing, and speech, thereby compromising overall oral health. Orthodontic treatment [2, 3] addresses these issues by correcting dental and jaw misalignments, with the dual aims of restoring function and enhancing aesthetics. Typically involving fixed or removable appliances, orthodontic treatment applies controlled forces to achieve an optimal occlusion. However, as a prolonged process, it often introduces challenges such as mucosal irritation, chewing difficulties, dietary restrictions, and temporary speech alterations. These discomforts may adversely affect patients’ emotional well-being during treatment [4].

Mental health is a critical determinant of orthodontic outcomes and management efficacy [5]. Orthodontic patients experiencing anxiety or depression are more likely to resist future treatment [6], Patients experiencing anxiety or depression may exhibit poorer compliance [7]. Given that psychological states tend to fluctuate throughout the treatment timeline [8], timely monitoring and tailored psychological support are essential for improving outcomes. However, without rigorous comparative designs, observed psychological changes may be misinterpreted. Current evidence presents two major limitations: first, a predominance of cross-sectional studies [8–10], which cannot model dynamic psychological trajectories; and second, a lack of adequately controlled longitudinal studies [11, 12], which weakens the evidence for attributing psychological changes specifically to orthodontic treatment, as opposed to natural variation or unmeasured confounders.

To this end, this study innovatively combines a propensity score matching (PSM) control group with an eight-stage longitudinal assessment. It aims to examine the association between orthodontic treatment and trajectories of mental health after controlling for key demographic confounding factors, and to explore differences in psychological adaptability between patients at different life stages (youth vs. maturity). The results will provide evidence-based insights for developing phased and population-specific psychological care pathways.

## Materials and methods

### Participants

#### Orthodontic group

Patients who commenced orthodontic treatment between March 2023 and October 2024 were enrolled using a convenience sampling approach. The inclusion criteria were as follows: (1) diagnosed with Angle Class I–III malocclusion; (2) aged between 18 and 59 years; (3) expected to undergo orthodontic treatment for more than 15 months, as determined through doctor-patient consultation; (4) no self-reported history of mental illness; (5) conscious and capable of normal communication; and (6) provided informed consent and were willing to participate in the study. The exclusion criteria included: (1) discontinuation of orthodontic treatment due to special circumstances; (2) requirement of orthognathic surgery; (3) refusal to complete the questionnaire or provision of incomplete responses; and (4) voluntary withdrawal from the study.

#### Control group

During the same period, patients receiving routine dental care (e.g., teeth cleaning, fillings) at the general dental clinic of the same hospital were recruited as controls. The inclusion criteria for this group were: (1) aged between 18 and 59 years; (2) no need or plan for orthodontic treatment; (3) absence of severe oral diseases (periodontitis less than stage III); and (4) no self-reported history of mental illness.

The two groups were matched using a 1:1 PSM procedure based on age (±3 years), gender, occupation, education level, marital status, and income level, with a caliper value set to 0.2. This study was approved by the Ethics Committee of Nantong Stomatological Hospital (Approval number: PJ 2024-022-01). The approval explicitly covers all participants involved in this research, including both the orthodontic treatment group and the non-orthodontic control group. Although the eligibility criteria for both groups specified an age range of 18–59 years, aiming to encompass a broad young and middle-aged population, the actual age range of participants who were successfully enrolled, completed the longitudinal follow-up, and formed the matched cohort was 18–42 years. This reflects the actual distribution of the clinical sample that consented to and adhered to the study protocol, which primarily comprised young and early middle-aged adults.

### Research tools

#### General information questionnaire

A self-designed general information questionnaire was used to collect data on the demographic and background characteristics of the patients, including gender, age, nature of workplace, marital status, education level, family per capita monthly income, and type of orthodontic appliance used.

#### Mental health scale

The mental health status of young and middle-aged orthodontic patients was assessed using the Chinese version of the Kessler 10 scale. The original Kessler 10 scale (referred to as ‘The Kessler Psychological Distress Scale’) was developed by Kessler et al. [13] at the University of Michigan and measures psychological distress. In 2008, Chinese scholars [14] validated the reliability and validity of the scale. The Chinese version demonstrated a Cronbach’s α coefficient of 0.801, indicating good internal consistency. The scale consists of 10 items scored on a five-point scale, with a total score ranging from 0 to 50. Mental health status is categorized as follows: (1) 10–15 points: good mental health; (2) 16–21 points: general mental health; (3) 22–29 points: poor mental health; and (4) 30–50 points: very poor mental health [15]. Higher scores indicate a worse mental health status. The scale is designed to assess psychological status over a 4-week interval. For this study, a 1-month interval was chosen for repeated measurements. The Cronbach’s α coefficient for this study’s sample was 0.833, confirming the reliability of the scale in this context.

#### Classification of malocclusion deformities

Using the Angle classification method, it is divided into three categories. Class I: The first molars of the maxilla and mandible are in a neutral relationship; Class II: The mesiobuccal cusp of the maxillary first molar occludes in front of the buccal groove of the mandibular first molar; Class III: The mesiobuccal cusp of the maxillary first molar occludes behind the buccal groove of the mandibular first molar.

#### Level of social support

This study utilized the Chinese version of the Social Support Rating Scale (SSRS), developed by Xiao [16], to evaluate the level of social support among the research participants. The scale comprises three dimensions: objective support, subjective support, and utilization of social support, encompassing a total of 10 items. Scoring procedures for the items are as follows: Items 1–4 and 8–10 are single-choice questions scored on a four-point Likert scale, where response options 1 through 4 correspond directly to scores 1 through 4. Item 5 consists of four sub-items (A, B, C, and D), each rated from 1 (‘no support’) to 4 (‘full support’), with the total score calculated as the sum of the scores across all sub-items. Items 6 and 7 are multiple-choice questions; ‘no source at all’ is assigned a score of 0, while each selected source under ‘the following sources’ contributes 1 point to the total score. A higher overall score indicates a greater level of perceived social support.

### Data collection and quality control

Mental health assessments were conducted at predetermined timepoints via telephone by trained orthodontic nurses. For the orthodontic group, questionnaires were administered at eight timepoints: T1 (treatment plan finalization), T2 (pre-appointment placement), T3 (1 month), T4 (3 months), T5 (6 months), T6 (9 months), T7 (12 months), and T8 (15 months). To capture core psychological changes while minimizing burden on the non-treatment group, the control group was assessed at three strategic timepoints: baseline upon enrollment (T0), the mid-treatment adaptation phase (concurrent with the orthodontic group’s T5), and at treatment completion (concurrent with T8). Each assessment took approximately 10 minutes. The Kessler-10 (K10) scale included three reverse-coded items to monitor response consistency. Participation was voluntary, and participants were instructed to respond truthfully. To ensure data quality, all research nurses received standardized training on the study protocol, the significance of each assessment point, and the neutral administration of the K10 and SSRS. Data were entered into Excel by one researcher and independently verified by two others to ensure accuracy.

### Statistical methods

Statistical analyses were performed using SPSS 25.0 (IBM, USA) and R 4.3.1. Continuous variables, which were confirmed to follow a normal distribution, are presented as mean ± standard deviation. Generalized estimating equations (GEE) with an exchangeable working correlation structure were employed as the primary method to analyze longitudinal mental health data, chosen for their robustness in handling correlated repeated measures and potential missing data. Robust (sandwich) estimators were used to calculate all standard errors (SE), ensuring valid inference even if the working correlation structure was misspecified. The primary model to test for differential trends between groups was specified as: Mental Health Score = β₀ + β₁ × Group + β₂ × Time + β₃ × (Group × Time), with β₃ testing the interaction effect. To control for demographic confounding, PSM was applied. Based on clinical relevance and literature review, core covariates were included in a 1:1 nearest-neighbor matching algorithm with a caliper width of 0.2. Balance between groups was assessed using the standardized mean difference (SMD); all covariates achieved an SMD < 0.1 after matching, indicating adequate balance. To ensure that the study design (especially with only three measurement points in the control group) had sufficient statistical power to detect clinically significant effects, a statistical power validation was conducted. Post-hoc power analysis was performed using G*Power 3.1 software. The parameters were set as follows: significance level α = 0.05 (two-tailed), sample size *N* = 248, number of groups = 2, number of measurements were 8 (orthodontic group), and 3 (control group), and a medium effect size (*f* = 0.25) was predicted. The analysis results showed that the statistical power to test the interaction between group and time reached 92.3%, which exceeds the conventional threshold of 80%. This suggests that the study had sufficient statistical power to detect a medium effect size of the group-by-time interaction, even with the asymmetrical measurement schedule between groups. It should be noted that this is a post-hoc analysis, and its value is primarily descriptive, confirming that the observed data were sensitive enough to detect effects of the anticipated magnitude.

## Results

### General characteristics of the 154 orthodontic patients

The study included 154 orthodontic patients aged between 18 and 42 years, with a mean age of 29.33 ± 7.54 years. Among them, 89 were female, and 65 were male. Occupational distribution included 92 individuals working in enterprises and institutions, 21 students, and 41 freelancers. Regarding marital status, 89 patients were married, and 65 were unmarried. Educational levels were categorized as follows: 24 individuals had a high school education or lower, 31 had completed junior college, and 105 held a bachelor’s degree or higher. Family per capita monthly income was distributed as follows: 21 individuals reported an income of <5,000 RMB, 98 reported between 5,000 and 10,000 RMB, and 35 reported >10,000 RMB. In terms of orthodontic methods, 91 patients underwent invisible correction, while 63 opted for fixed correction. The total score of social support ranged from 22 to 56 (38.21 ± 6.33), as shown in Table 1.

**Table 1:** General information of orthodontic patients (*N* = 154).

Characteristic	Group	Number of cases	Percentage
Age (years)	18~26	72	46.8
	27~42	82	53.2
Gender	Female	89	57.8
	Male	65	42.2
Occupation	Enterprise and public institution	92	59.7
	Student	21	13.6
	Freelancer	41	26.6
Marital status	Married	89	57.8
	Unmarried	65	42.2
Education background	High school and below	24	15.6
	Junior college	31	20.1
	Bachelor’s degree or above	99	64.3
Monthly per capita income of a household (RMB)	< 5,000	21	13.6
	5,000~10,000	98	63.7
	> 10,000	35	22.7
In terms of orthodontic methods	Invisible correction	91	59.1
	Fixed correction	63	40.9
Classification of malocclusion deformities	Class I (Neutral)	58	37.7
	Class II (distal)	72	46.7
	Class III (Mesial)	24	15.6

### Mental health scores of young and middle-aged orthodontic patients at different time points

GEE analysis revealed a significant temporal trend in the mental health status of young and middle-aged orthodontic patients during treatment (Wald χ² = 304.51, *p* < 0.001). From the determination of the orthodontic plan to 9 months into treatment, the K10 scores of the patients were all above 15 points, indicating a sub-healthy mental state. By 12–15 months of treatment, the scores dropped below 15 points, suggesting a recovery to a good mental health status. The detailed scores at each time point are presented in Table 2.

**Table 2:** Mental health scores of young and middle-aged orthodontic patients at each time point (*N* = 154).

Node of time	Minimum value	Maximum value	Mental health score
T1	9	24	19.41 ± 3.19
T2	11	26	20.11 ± 3.02
T3	8	23	19.01 ± 2.89
T4	6	22	17.92 ± 2.84
T5	10	18	15.25 ± 2.66
T6	9	19	15.27 ± 2.49
T7	11	20	14.87 ± 2.45
T8	12	19	14.72 ± 2.31

### Temporal variations in mental health status among young and middle-aged orthodontic patients

**Figure 1:**
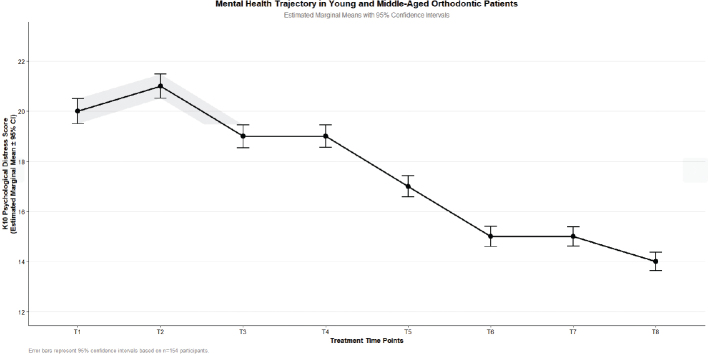
Change curve of mental health status in young and middle-aged orthodontic patients.

The post hoc pairwise comparison analysis results based on GEE (see Table 3) indicate that the mental health status of orthodontic patients in the middle-aged and young age groups shows significant dynamic changes over time. Specifically, there is no statistically significant difference in scores from T1 (the day of determining the treatment plan) to T2 (before wearing the orthodontic appliance) (*p* > 0.05). From T2 to T5 (6 months of treatment), the mental health scores show a significant downward trend (all pairwise comparisons *p* < 0.05). However, from T5 to the end of treatment (T8, 15 months), there is no statistically significant difference in scores between each time point (*p* > 0.05), indicating that the mental health status enters a stable plateau period. This change trend is shown in Figure 1.

**Table 3:** Pairwise comparison of mental health scores of young and middle-aged orthodontic patients across time points (*N* = 154).

Node 1	Node 2	Mean difference	Standard error	95% CI	*P*
T_1_	T_2_	–0.08	0.06	–0.20 to 0.04	0.201
	T_3_	1.134	0.42	0.29 to 1.98	0.009
	T_4_	2.672	0.81	1.08 to 4.26	< 0.001
	T_5_	5.011	1.52	2.03 to 7.99	< 0.001
	T_6_	5.329	1.62	2.15 to 8.50	< 0.001
	T_7_	5.761	1.75	2.33 to 9.19	< 0.001
	T_8_	5.813	1.77	2.34 to 9.28	< 0.001
T_2_	T_3_	1.142	0.47	0.22 to 2.06	0.016
	T_4_	2.712	0.82	1.10 to 4.32	< 0.001
	T_5_	5.023	1.53	2.03 to 8.01	< 0.001
	T_6_	5.419	1.65	2.19 to 8.65	< 0.001
	T_7_	5.798	1.76	2.35 to 9.25	< 0.001
	T_8_	5.934	1.80	2.41 to 9.46	< 0.001
T_3_	T_4_	1.954	0.59	0.80 to 3.11	< 0.001
	T_5_	3.232	0.98	1.31 to 5.15	< 0.001
	T_6_	3.541	1.08	1.42 to 5.66	< 0.001
	T_7_	4.112	1.25	1.66 to 6.56	< 0.001
	T_8_	4.324	1.31	1.76 to 6.89	< 0.001
T_4_	T_5_	2.012	0.61	0.82 to 3.21	< 0.001
	T_6_	2.432	0.74	0.98 to 3.88	< 0.001
	T_7_	3.141	0.95	1.28 to 5.00	< 0.001
	T_8_	3.211	0.98	1.29 to 5.13	< 0.001
T_5_	T_6_	0.281	0.43	–0.56 to 1.12	0.511
	T_7_	0.889	1.53	–2.11 to 3.89	0.561
	T_8_	0.911	0.55	–0.17 to 1.99	0.097
T_6_	T_7_	0.711	0.68	–0.62 to 2.04	0.301
	T_8_	0.822	0.78	–0.71 to 2.35	0.287
T_7_	T_8_	0.211	1.41	–2.55 to 2.97	0.882

Note: CI: confidence interval. Pairwise comparisons were performed using the estimated marginal means from the generalized estimating equations (GEE) model with a Bonferroni adjustment for multiple comparisons.

### Propensity score matching results

**Figure 2:**
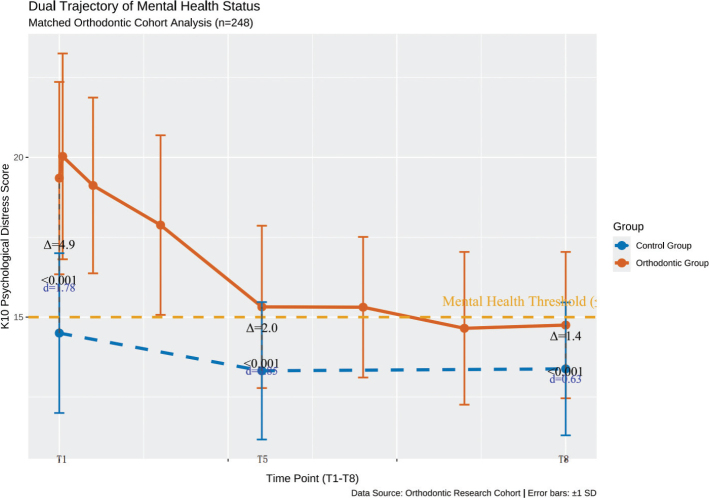
Change curve of mental health level between orthodontic group and control group after matching.

Through PSM, a 1:1 nearest neighbor matching method was employed to match patients in the orthodontic group (*n* = 154) with those from the control group candidate pool (*n* = 210). A total of 124 matched pairs were successfully identified, resulting in a matching rate of 80.5%. The final sample size after matching comprised 248 cases. Following matching, all SMDs for covariates were less than 0.1 (ranging from 0.02 to 0.07), indicating adequate balance in demographic characteristics between the two groups. For detailed matching results, refer to Table 4. The analysis revealed a significant change over time in the orthodontic group (*F* = 228.7, *p* < 0.001), whereas no significant variation was observed in the control group (*F* = 0.92, *p* = 0.401). Additionally, a significant interaction effect was detected (β = –0.41, *p* < 0.001), suggesting that the observed psychological trajectory was associated with the orthodontic treatment process. Table 5 and Figure 2 presents the mental health scores of both the orthodontic and control groups before and after matching. The effect size (Cohen’s *d*) of the inter-group comparison revealed the degree of difference in mental health at different treatment stages. At the baseline (T1), there was a large effect size difference between the orthodontic group and the control group (*d* = 1.78), which corresponded to a significant increase in psychological burden among patients, potentially reflecting anticipatory anxiety before treatment. At the mid-treatment stage (T5, 6 months) and the late treatment stage (T8, 15 months), although the effect size of the inter-group differences decreased, they still reached a moderate effect (*d* = 0.85) and a borderline effect from moderate to large (*d* = 0.63), respectively. According to Cohen’s benchmarks, *d* > 0.8 is generally considered to have clinical significance. Therefore, the observed association between orthodontic treatment and mental health changes is not only statistically significant but also potentially clinically meaningful.

**Table 4:** Characteristics of the matched and unmatched cohorts (*N* = 248).

Variate	Orthodontic group after matching (*n* = 124)	Control group (*n* = 124)	SMD
Age (years)	29.8 ± 7.3	30.1 ± 6.9	0.05
Gender (Female)	58.1%	57.7%	0.03
Bachelor’s degree or above	63.7%	62.9%	0.07
Married	58.9%	59.3%	0.04
Monthly per capita income of a household > 10,000	35.5%	34.7%	0.06

SMD: standardized mean difference.

**Table 5:** Time series analysis of mental health status in matched cohort (*N* = 248).

Time node	Clinical stage	Orthodontic group (*n* = 124)	Control group (*n* = 124)	Inter-group difference (Δ)	Effect size (*d*)	*P*
T1	Date for determining the plan	19.35 ± 3.01	14.50 ± 2.50	+4.85	1.78	< 0.001
T2	One day before wearing orthodontic appliances	20.03 ± 3.22	-	-	-	-
T3	One month of treatment	19.12 ± 2.75	-	-	-	-
T4	3-months of treatment	17.88 ± 2.81	-	-	-	-
T5	6-months of treatment	15.32 ± 2.54	13.32 ± 2.15	+2.00	0.85	< 0.001
T6	9-months of treatment	15.31 ± 2.20	-	-	-	-
T7	12-month treatment	14.65 ± 2.39	-	-	-	-
T8	15-months of treatment	14.75 ± 2.29	13.38 ± 2.08	1.37	0.63	< 0.001

Calculation of effect sized: Cohen’s *d* = (M_1_ – M_2_) / SD_poole_d.

### Analysis of influencing factors on the mental health of young and middle-aged orthodontic patients

Using the mental health score of the patients as the dependent variable and general information as the independent variable, GEE analysis identified several factors significantly affecting mental health status. These factors include gender, occupation, family per capita monthly income, education level, and marital status. The differences were statistically significant (*p* < 0.05), as shown in Table 6.

**Table 6:** Generalized estimating equation analysis of mental health in young and middle-aged orthodontic patients (*N* = 154).

Variables	β	SE	*Z*	*P*
Gender				
Female	6.12	1.28	4.78	< 0.001
Occupations				
Students vs. Enterprises and Public Institutions	2.18	0.94	2.32	0.020
Freelancing vs. Enterprises and public institutions	1.52	0.82	1.85	0.064
Monthly household income per capita (RMB)				
5,000–10,000 vs < 5,000	–1.85	0.07	–26.43	< 0.001
> 10,000 vs < 5,000	–3.51	0.17	–20.65	< 0.001
Level of education				
Junior college vs. High school and below	–1.32	0.30	–4.40	< 0.001
Bachelor’s degree or higher	–1.89	0.53	–3.57	< 0.001
Marital status				
Unmarried VS Married	3.05	1.18	2.58	0.010
Classification of malocclusion deformities				
Class II vs Class I	0.78	0.45	1.73	0.083
Class III vs Class I	1.23	0.52	2.37	0.018
Total score of social support	–0.35	0.07	–5.00	< 0.001

Note: Independent variable assignment, gender: male = 1, female = 2; Enterprises and institutions were used as references to set dummy variables. Household per capita monthly income < 5,000 RMB was the dummy variable. The dummy variable was set with an educational level of technical secondary school or below. Marital status: married = 1, unmarried = 2. Dummy variables were created to classify malocclusion deformities, using Class I as the reference category.

### Subgroup analysis by age

**Figure 3:**
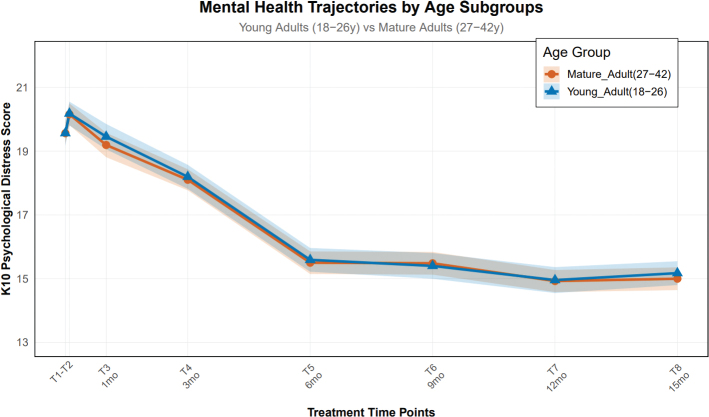
The mental health trajectories of orthodontic patients at different age stages.

To deeply explore the potential impact of age on the mental health trajectory, we divided the sample into two subgroups: young adulthood (18–26 years old, *n* = 72) and mature adulthood (27–42 years old, *n* = 82). Baseline comparisons showed that the two groups were balanced in key variables such as gender, occupation, income, educational level, and marital status (all SMD < 0.1), demonstrating good comparability. Linear mixed model analysis revealed that the interaction effect between group and time point was not significant (*F*(7, 1,078) = 0.89, *p* = 0.512), indicating that the mental health change trends during orthodontic treatment were consistent between the young and mature groups. As shown in Figure 3, although the mature adult group scored slightly higher at each time point than the young adult group, the differences between the groups were not statistically significant (*p* > 0.05 for all time points). This result indicates that age is not a key factor associated with the mental health trajectory in this population aged 18–42, and the psychological adaptation process observed during orthodontic treatment is universal among adult patients of different ages.

## Discussion

This study, utilizing a balanced cohort constructed by propensity score matching, for the first time describes a ‘stress-adaptation-stabilization’ trajectory of mental health in young and middle-aged orthodontic patients. Notably, psychological distress peaked before appliance placement, suggesting that anticipatory anxiety may be a critical initial stressor, consistent with prior research on pre-treatment anxiety [17]. This finding emphasizes the potential value of psychological support before treatment initiation. The core contribution of this study is the identification of a robust longitudinal association between the orthodontic treatment process and changes in mental health, after controlling for key demographic confounders. The initial decline in distress (T1–T4) may relate to physiological adaptation, while later stability (T5–T8) coincides with growing treatment familiarity and anticipated aesthetic improvements [10, 18]. Importantly, mean scores remained above the clinical cut-off [15] for much of the treatment, underscoring the chronic nature of this stressor.

Our findings move beyond single factors to outline a multi-factorial profile. Key risk factors included female gender, student status, unmarried status, and severe (Class III) malocclusion, whereas higher socioeconomic status and stronger social support were protective factors. This aligns with existing evidence: female patients report higher treatment-related anxiety [19, 20]; students face unique stressors [21, 22]; and social support is a well-established buffer against stress [23, 24]. The significant role of malocclusion severity, even in non-surgical cases, highlights the functional and psychosocial burden of severe malocclusion [25, 26].

An important finding was the lack of a significant effect of age on the psychological trajectory [27, 28]. This suggests that psychological adaptation processes in early- to mid-adulthood may be similar across this age range, and that socio-psychological factors (e.g., marital or student status) are more critical for risk stratification than age alone. These results argue for a stratified approach to psychological care in orthodontics. High-risk patients (e.g., unmarried female students with Class III malocclusion) may benefit from early, targeted support. Routine assessment of social support and malocclusion-related concerns should be considered. Interventions could be phased, targeting anticipatory anxiety pre-treatment and adaptation challenges during early treatment.

This study has several limitations. First, the observational design demonstrates association, not causation. Second, the use of telephone-administered surveys may introduce recall and social desirability biases. Third, as the control group was assessed at only three timepoints, this may limit the precision and interpretability of the longitudinal statistical model. While three points can indicate linear or quadratic trends, they are insufficient to reliably fit more complex, non-linear phase transitions, which could affect the granularity of the trajectory model. Fourth, the propensity score matching model could not adjust for all clinical variables, leaving potential for residual confounding. Despite these limitations, the findings provide an important preliminary framework for dynamic psychological intervention in clinical practice. Future research should employ intensive longitudinal designs with more frequent assessments across the entire treatment cycle for all participants, including controls. This will allow for a more precise characterization of the dynamic process of psychological change and generate a high-resolution, data-driven ‘map’ to guide the timing of personalized interventions.

## Conclusion

This longitudinal study demonstrates that the mental health status of young and middle-aged orthodontic patients exhibits a phased improvement trajectory from the initial formulation of the treatment plan to 15 months into orthodontic therapy. This developmental pattern is associated with various sociodemographic factors, including gender, occupational role, economic status, educational background, and marital status. Accordingly, it is recommended that mental health screenings be integrated at critical stages of treatment, such as the initial appliance placement phase, the functional adaptation period, and the aesthetic refinement stage. For high-risk populations – particularly unmarried women and individuals with limited financial resources – clinical interventions should incorporate enhanced social support systems. Furthermore, communication strategies should be tailored to align with patients’ cultural contexts and individual treatment expectations. It is important to emphasize that this study, as an observational one, mainly contributes to revealing a robust longitudinal association between orthodontic treatment and mental health trajectories. Despite efforts to control for known confounding factors through propensity score matching and sensitivity analysis, which enhanced the credibility of the association between ‘treatment’ and ‘outcome’, the possibility of unmeasured confounding factors cannot be completely ruled out. Therefore, the findings of this study provide strong evidence in support of the notion that ‘orthodontic treatment may be an important influencing factor for mental health’, but the exact causal relationship still requires final confirmation through future randomized controlled trials or quasi-experimental designs.
